# Oral Mesotherapy as an Adjunctive Approach for the Management of Localized Periodontitis: A Case Report

**DOI:** 10.7759/cureus.115702

**Published:** 2026-09-03

**Authors:** Ruhee L Chawla, Shreya V Bhole, Sunil Ronad, Sakshi S Awaghad, Vijay Mehetre

**Affiliations:** 1 Department of Periodontology, Jawahar Medical Foundation's Annasaheb Chudaman Patil Memorial Dental College, Dhule, IND; 2 Department of Prosthodontics, Jawahar Medical Foundation's Annasaheb Chudaman Patil Memorial Dental College, Dhule, IND; 3 Department of Periodontology, Shree Dental Clinic and Implant Centre, Aurangabad, IND

**Keywords:** mesotherapy, periodontal pocket, periodontitis, platelet-rich fibrin, scaling and root planing

## Abstract

Periodontitis is a chronic inflammatory disease characterized by progressive destruction of the supporting structures of the teeth. While conventional therapies such as scaling and root planing (SRP) remain the cornerstone of treatment, their ability to restore previously lost periodontal attachment may be limited. Injectable platelet-rich fibrin (I-PRF), a second-generation autologous platelet concentrate, has emerged as a promising adjunct owing to its sustained release of growth factors that promote angiogenesis, fibroblast proliferation, and tissue repair. Oral mesotherapy, a minimally invasive technique involving targeted intratissue delivery, offers an innovative approach to administer I-PRF directly to the affected periodontal tissues. This case report describes a 52-year-old female diagnosed with localized Stage II, Grade B periodontitis currently unstable affecting the upper incisor region. Following SRP, the patient underwent two sessions of oral mesotherapy with I-PRF at intervals of one week. Comprehensive baseline laboratory investigations confirmed the systemic health and suitability of autologous preparation. Clinical parameters, including probing pocket depth (PPD), clinical attachment level (CAL), gingival and plaque indices, and bleeding on probing (BOP), were recorded at baseline and three months after treatment. Notable improvements were observed: PPD reduced from 5 mm to 2-3 mm, CAL improved, and BOP decreased to 10%. No adverse events were observed. This case demonstrates that oral mesotherapy with I-PRF was well tolerated and was associated with favorable clinical periodontal changes when used as an adjunct to conventional periodontal therapy for managing localized periodontitis. It is associated with faster wound healing and may enhance treatment outcomes beyond those of conventional modalities. Further controlled clinical studies are needed to validate and standardize this approach.

## Introduction

Periodontitis is a widespread inflammatory disease that affects the supporting structures of teeth, leading to progressive attachment loss, alveolar bone destruction, and, in advanced cases, tooth mobility and loss [1]. Globally, it affects 20-50% of adults, with severe forms affecting 10-15% of the population. In India, the prevalence of mild-to-moderate periodontitis is estimated at 26.2% and severe periodontitis at 19% [2,3]. The condition is driven by microbial biofilms, host immune responses, and environmental factors, posing significant challenges to treatment due to persistent inflammation and the limited regenerative capacity of periodontal tissues [4]. Conventional treatment modalities, such as scaling and root planing (SRP), adjunctive antimicrobial therapy, and occasionally surgical interventions, often yield suboptimal outcomes in mild-to-moderate cases, particularly when restoration of previously lost periodontal attachment is desired [4,5].

Recent advances in periodontal therapy have focused on regenerative approaches to address these limitations. Injectable platelet-rich fibrin (I-PRF), a second-generation platelet concentrate, has emerged as a promising adjunctive therapy owing to its ability to release growth factors such as platelet-derived growth factor (PDGF) and transforming growth factor-beta (TGF-β) [6]. These growth factors promote angiogenesis, fibroblast proliferation, and collagen synthesis, facilitating periodontal tissue repair [6,7].

Oral mesotherapy, a minimally invasive technique involving targeted intratissue injection of bioactive agents into gingival tissues, provides an innovative delivery method for I-PRF, ensuring localized and sustained release of regenerative factors at the site of inflammation [8]. Mesotherapy, alternatively referred to as local intratissue therapy, is a method whereby pharmacological agents are introduced into the dermal layer of the skin. The administration of drugs within the dermis results in the formation of a microdeposit, from which these agents gradually permeate into adjacent tissues [9]. Using this methodology, a drug-sparing effect is achieved in comparison to the systemic administration route, concomitantly leading to a reduction in the overall dosage of pharmaceutical agents [10]. This technique has been previously used in the management of gingival hyperpigmentation and has shown promising results [11,12].

Studies have shown that I-PRF, when used with SRP, significantly reduces the probing pocket depth (PPD) and improves clinical attachment level (CAL) in patients with periodontitis [13,14]. I-PRF mesotherapy was selected as a minimally invasive adjunct because of its potential biological benefits and localized delivery while avoiding the need for surgical intervention in this localized lesion. However, to date, I-PRF has never been used as an oral mesotherapy for the treatment of mild-to-moderate periodontal pockets. This case report demonstrates the successful delivery of oral mesotherapy with I-PRF in the management of localized periodontitis, demonstrating its potential as an effective therapeutic modality.

## Case presentation

A 52-year-old female presented to the Department of Periodontics with the chief complaint of bleeding gums and an urge to manipulate the gingiva with a toothpick, along with sensitivity to cold. The patient had undergone SRP two years before, which provided only temporary relief. She maintained regular toothbrushing but struggled with consistent oral hygiene. The patient had no systemic conditions such as diabetes or cardiovascular disease, no history of tobacco use, and no vital signs within normal limits (blood pressure, 118/78 mmHg; pulse, 72 bpm).

Clinical examination

Intraoral examination revealed that bleeding on probing (BOP) was positive at teeth #11 and #12. The PPD was measured at 5 mm on the mesial aspect of #12, with a CAL of 5 mm, with no gingival recession. The gingival index (GI) score was 2, indicating moderate inflammation with BOP observed in 30% of the anterior maxillary sites. The plaque index (PI) was 3, reflecting moderate plaque accumulation (Figure 1A). A digital intraoral periapical radiograph confirmed 31% horizontal bone loss in #12 (Figure 1B). Based on the available periodontal charting (Table 1) and radiographic findings, the case was classified according to the 2018 American Academy of Periodontology and the European Federation of Periodontology (AAP/EFP) periodontal disease classification; the case was diagnosed as localized Stage II, Grade B, periodontitis currently unstable [1].

**Figure 1:**
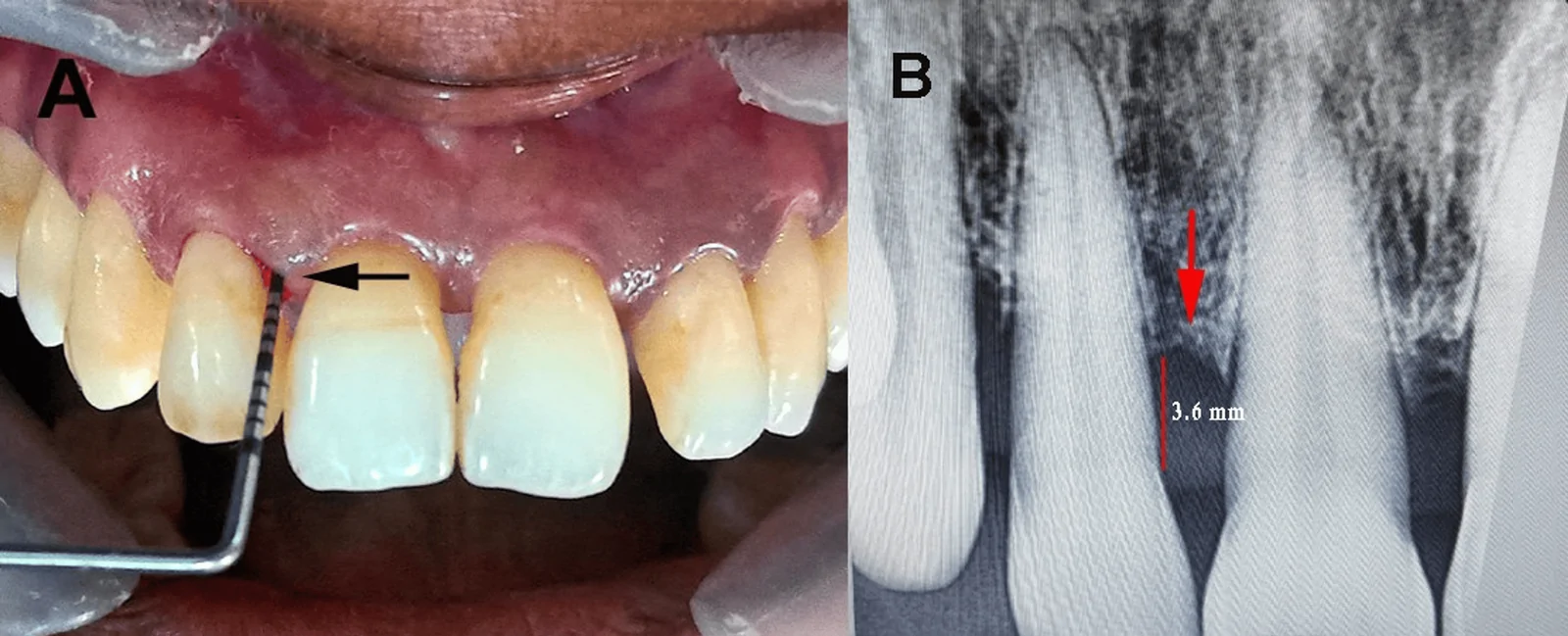
(A) Preoperative probing pocket depth of 5 mm (black arrow) between maxillary central and lateral incisor, and (B) preoperative digital IOPA showing alveolar bone loss of 3.6 mm (red arrow) between maxillary central and lateral incisor. The UNC-15 probe is used to measure the probing pocket depth. IOPA, intraoral periapical radiograph.

**Table 1:** Periodontal charting for the patient BOP, bleeding on probing; CAL, clinical attachment level; PPD, periodontal pocket depth.

Parameter	Baseline
Plaque index	3
Gingival index	2
BOP – anterior maxilla	30%
Maximum documented PPD	4-5 mm
Maximum documented CAL	4-5 mm
Gingival recession at #12 mesial	0 mm
Tooth mobility	No
Furcation involvement	No
Smoking status	No
Diabetes/HbA1c	No
Tooth loss attributable to periodontitis	No
Bone loss/age ratio	0.59

Treatment protocol

After obtaining informed consent, the patient underwent full-mouth SRP two weeks before initiating oral mesotherapy to reduce the microbial load and optimize the periodontal environment. A comprehensive panel of blood investigations was conducted before treatment initiation to ensure the safety and efficacy of oral mesotherapy with I-PRF. A complete blood count was performed to assess hemoglobin, red blood cell, white blood cell, and platelet counts. Platelet counts were within the normal range (150,000-450,000/µL), and there was no evidence of anemia or systemic infection. A coagulation profile, including prothrombin time, activated partial thromboplastin time, and international normalized ratio, was evaluated to verify normal clotting function, which is critical for fibrin matrix formation in I-PRF. Fasting blood glucose (FBG) and glycated hemoglobin (HbA1c) tests were carried out to exclude undiagnosed diabetes, which could exacerbate periodontitis or hinder healing outcomes, with results confirming normal glycemic status (FBG <100 mg/dL, HbA1c <5.7%). Serum C-reactive protein levels were measured to assess systemic inflammation, with values within the normal range (<10 mg/L), indicating the absence of underlying inflammatory conditions. Additionally, liver function tests, including alanine aminotransferase, aspartate aminotransferase, alkaline phosphatase, and total bilirubin, and renal function tests, including serum creatinine, blood urea nitrogen, and estimated glomerular filtration rate, were conducted to ensure optimal systemic health for tissue regeneration and rule out liver or kidney dysfunction that could affect I-PRF quality or healing (Table 2). All investigations performed before baseline measurements on day 0 confirmed the patient’s suitability for I-PRF therapy, aligning with her systemically healthy status and contributing to the absence of adverse effects during the three-month follow-up period.

**Table 2:** Laboratory investigation of patient blood profile with normal range of test value

Investigation	Patient value	Reference range
C-reactive protein	2.8 mg/L	<10 mg/L
Alanine aminotransferase	21 U/L	7-35 U/L
Aspartate aminotransferase	19 U/L	8-33 U/L
Alkaline phosphatase	82 U/L	44-147 U/L
Total bilirubin	0.7 mg/dL	0.1-1.2 mg/dL
Serum creatinine	0.8 mg/dL	0.5-1.1 mg/dL
Blood urea nitrogen	14 mg/dL	7-20 mg/dL
Estimated glomerular filtration rate	88 mL/min/1.73 m²	≥60 mL/min/1.73 m²

I-PRF was prepared using the IntraSpin system (Intra-Lock International, Boca Raton, FL, USA). A 10 mL sample of autologous venous blood was collected from the antecubital vein and centrifuged at 700 rpm (60 × g) for 3 minutes at room temperature without an anticoagulant, following the protocol of Miron et al. [6]. The supernatant containing I-PRF was collected for injection.

Before each session, topical lignocaine gel (Xylocaine® 2% Gel, AstraZeneca, Cambridge, UK) was applied to the keratinized gingival tissues of #11, #12 for 2-3 minutes to minimize surface discomfort, with 0.5 mL of 2% lignocaine (Lignospan® Special, Septodont, Saint-Maur-des-Fossés, France) administered via infiltration anesthesia on the first visit (day 0) to the buccal and lingual aspects for deeper injection comfort. The subsequent visit (day 7) relied on topical anesthesia unless discomfort required additional infiltration. Oral mesotherapy was performed using a Dispovan Insulin syringe 30-gauge needle (Hindustan Syringes & Medical Devices, Faridabad, Haryana), 0.2 mL was injected at each site into the gingival connective tissue (at the base of periodontal pocket), 2 mm apical to the gingival margin, at the labial and lingual aspects of #11 and #12 (resulting in a total administered volume of 0.8 mL per session), with the needle inserted perpendicular to the gingival tissues (bevel upwards) until slight tissue blanching was observed, ensuring precise delivery (Figure 2). The second injection was performed on day 7 following baseline measurements on day 0. After treatment, the patient used 0.12% chlorhexidine mouthwash (PerioGard®, Colgate-Palmolive, New York, NY, USA) twice daily for two weeks and was instructed to maintain meticulous oral hygiene with twice-daily brushing and interdental cleaning.

**Figure 2:**
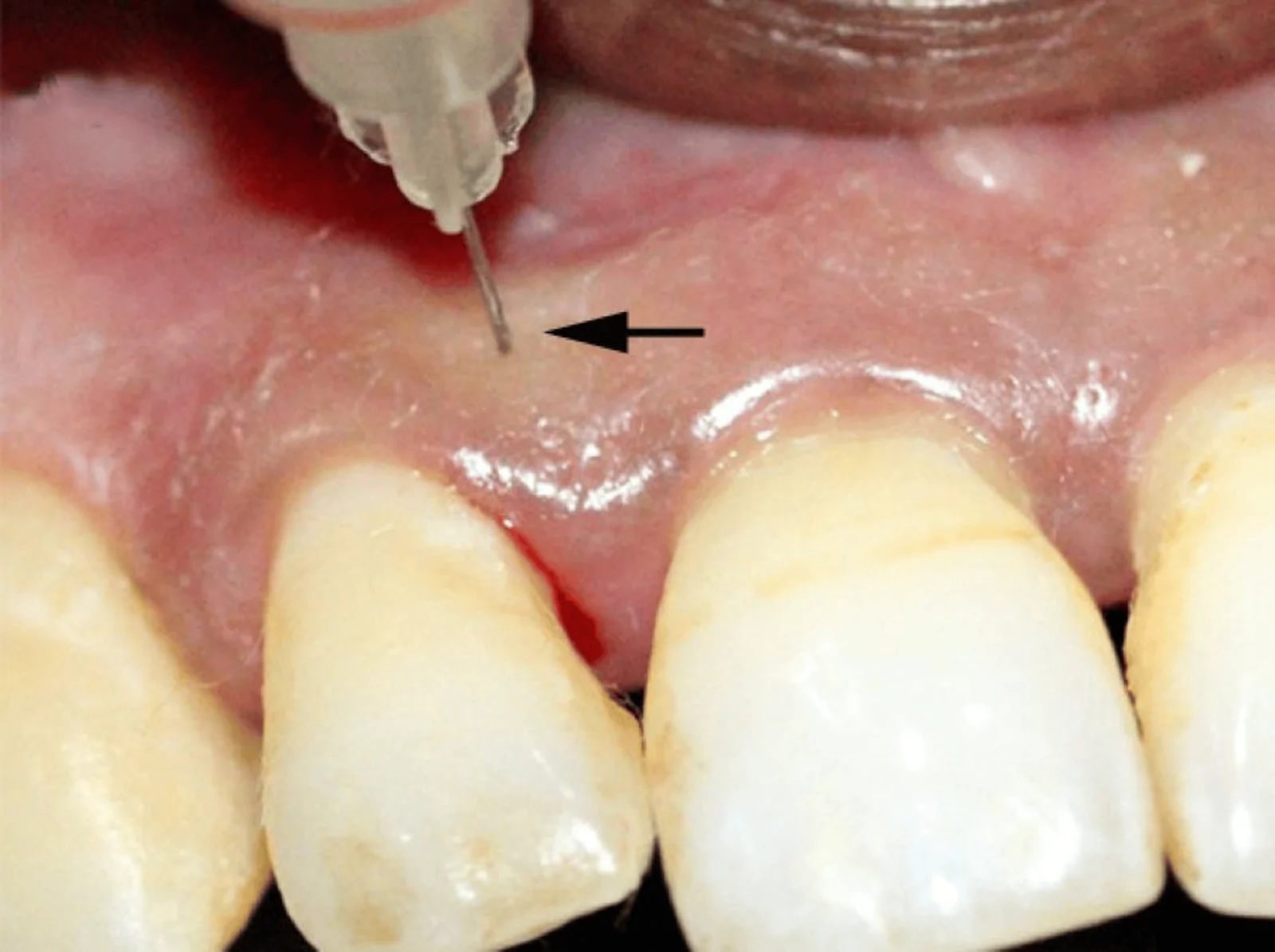
Mesotherapy injection of I-PRF at 2 mm below gingival margin at the base of pocket (black arrow) using insulin syringe I-PRF, injectable platelet-rich fibrin.

Follow-up and outcomes

Follow-up evaluations were performed regularly. At the three-month follow-up, marked clinical improvement was observed. PPD decreased from 5 mm to 2-3 mm in #11 and #12 (Table 3).

**Table 3:** Clinical periodontal measurements, including PPD and CAL CAL, clinical attachment level; PPD, periodontal pocket depth.

Tooth site	Baseline PPD (mm)	Baseline CAL (mm)	Three-month PPD (mm)	Three-month CAL (mm)
#11 -- distal	4	4	2	2
#12 -- mesial	5	5	3	3

The GI and PI scores were both reduced to one, reflecting minimal inflammation and plaque accumulation. BOP decreased to 10% of the sites in the anterior maxilla. The patient reported a substantial improvement in aesthetic satisfaction due to stabilized gingival margins (Figure 3). No adverse effects such as infection, swelling, or allergic reactions were observed during the follow-up period, indicating the safety and tolerability of the intervention.

**Figure 3:**
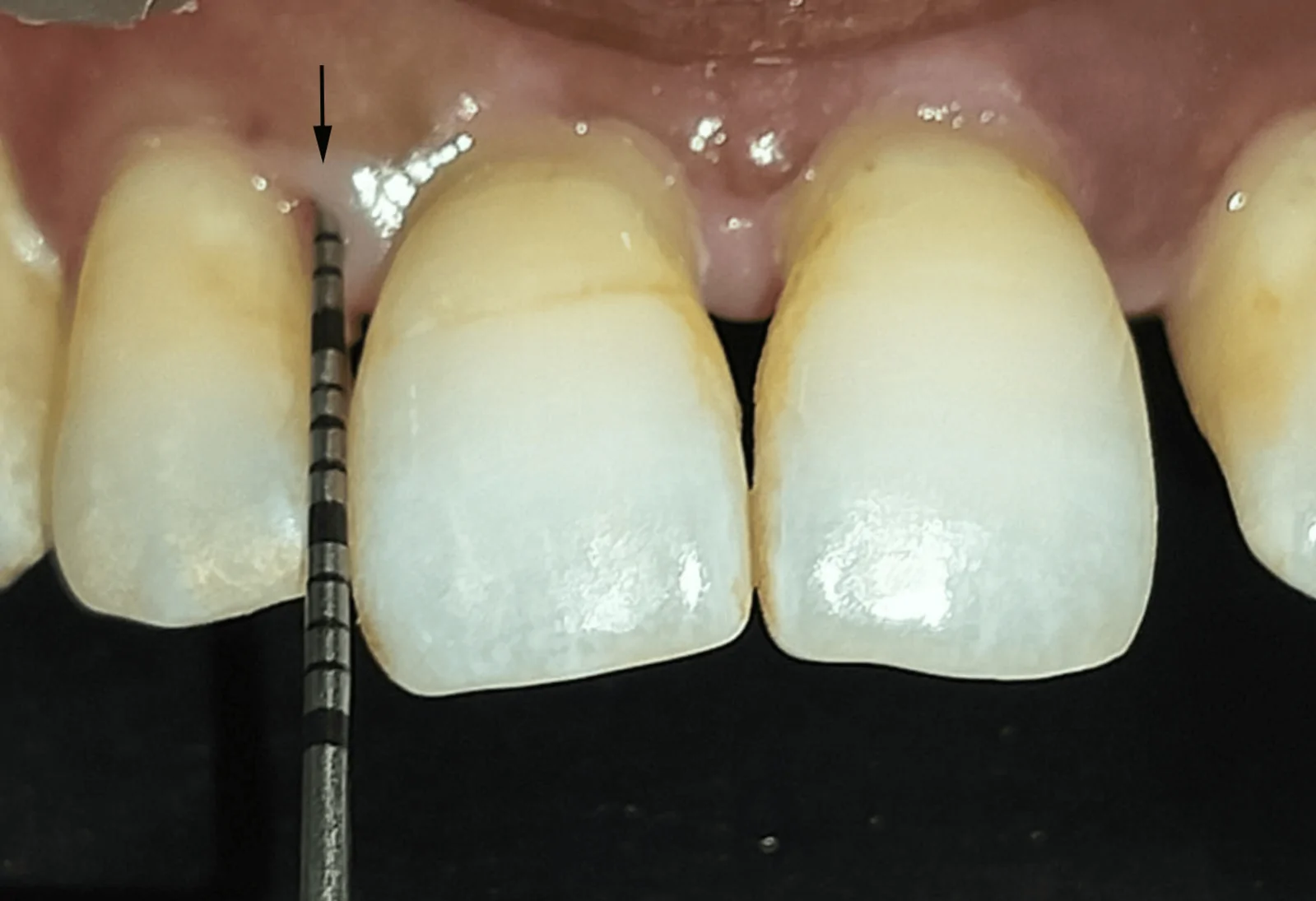
Postoperative follow-up at three-month showed probing pocket depth of 2-3 mm (black arrow) between right maxillary central and right lateral incisor. The UNC-15 probe is used to measure the probing pocket depth.

## Discussion

Periodontitis management remains a significant clinical challenge, particularly in cases requiring not only resolution of inflammation but also healing of lost periodontal structures [3]. Traditional therapeutic modalities, including SRP and optimal surgical interventions, often demonstrate repair capacity to some extent, particularly in mild-to-moderate cases [4]. This case report illustrates the novel and successful application of oral mesotherapy with I-PRF as a minimally invasive adjunct to conventional periodontal therapy for the management of localized Stage II, Grade B periodontitis currently unstable.

Manasa et al. [15] and Ozsagir et al. [16] reported increases in gingival thickness following I-PRF therapy compared with baseline measurements, with a 29% increase after six months and a 0.16-mm increase after three months, respectively. In clinical trials, the adjunctive use of I-PRF with SRP resulted in significantly greater reductions in PPD and CAL gains than SRP alone [17,18].

The similarities observed in the findings can be attributed to various factors. Elevated levels of growth factors, platelets, leukocytes, and cytokines present in I-PRF confer numerous benefits during the process of periodontal tissue repair. Additionally, I-PRF enhances fibroblast migration and promotes the upregulation of PDGF, type-1 collagen, and transforming growth factor [15]. Moreover, the three-dimensional fibrin matrix serves as a biological scaffold, facilitating the aggregation of adherent cells at the site of intervention [18]. Adhikary et al. [19] reported a significant increase in keratinized tissue width at three and six months of follow-up compared with baseline, which could be attributed to the gradual release of high concentrations of growth factors important for periodontal healing. 

However, the delivery of biological agents plays a crucial role in determining therapeutic efficacy. In this case, oral mesotherapy, a technique borrowed from dermatological and cosmetic medicine, was employed to deliver I-PRF directly into the gingival connective tissue [9]. Mesotherapy allows localized and controlled delivery of bioactive agents, forming a drug reservoir in the dermal or subepithelial space that gradually diffuses into surrounding tissues, thereby sustaining therapeutic action while minimizing systemic exposure [9,10]. In dermatology, this technique has been effectively used for skin rejuvenation and alopecia, and recent dental literature has started exploring its role in treating conditions such as gingival hyperpigmentation and recurrent aphthous ulcers [11,12]. The current case marks a novel application of this delivery system in periodontics.

The observed clinical improvements, including reduction of PPD from 4-5 mm to 2-3 mm and stabilization of gingival recession, support the hypothesis that the localized, minimally invasive delivery of I-PRF enhances periodontal healing. These findings are in line with those reported by Jia et al. [20], who demonstrated that the sustained release of growth factors from I-PRF can persist for up to 10-14 days, supporting tissue healing beyond the acute phase of inflammation.

The use of 0.12% chlorhexidine for two weeks after treatment and reinforcement of oral hygiene represent additional potential confounders. Both measures may have contributed to reductions in plaque accumulation, gingival inflammation, and BOP independently of the I-PRF intervention. Because this was a single case without a control condition, the relative contribution of I-PRF, SRP, and chlorhexidine, and improved oral hygiene cannot be separated. Future controlled studies should standardize or account for these co-interventions when evaluating the independent clinical effect of I-PRF mesotherapy.

Furthermore, the safety of I-PRF and oral mesotherapy was demonstrated in this case. No adverse effects were observed, aligning with the autologous nature of I-PRF and the well-tolerated, minimally traumatic mesotherapy technique. Blood investigations before therapy ensured systemic suitability and eliminated confounding systemic inflammatory- or coagulation-related variables that could impair healing. This step is supported by the recommendations of Miron et al. [7], who emphasized the importance of patient systemic health for optimal I-PRF efficacy.

An important limitation of this case is the absence of a separate control or observation period following initial periodontal therapy. Full-mouth SRP was performed two weeks before I-PRF mesotherapy, and oral hygiene instructions were reinforced throughout treatment. Therefore, the clinical improvements observed at three months cannot be attributed solely to I-PRF mesotherapy.

Although this case report demonstrates encouraging outcomes with the use of oral mesotherapy and I-PRF in periodontal therapy, further research is essential to validate these findings. Future studies should include randomized controlled trials with larger sample sizes to compare this technique with conventional attachment regenerative methods and assess parameters such as probing depth reduction, CAL gain, and radiographic bone fill. Long-term follow-up studies are also recommended to evaluate the stability and durability of observed clinical improvements. Standardization of injection protocols, such as volume, frequency, and depth of delivery, should be explored to optimize the treatment outcomes. Investigating the application of this technique at different stages and grades of periodontitis, as well as in medically compromised patients, may further expand its clinical utility.

## Conclusions

In conclusion, oral mesotherapy with I-PRF was well tolerated and associated with favorable clinical periodontal changes in this case of localized periodontitis. The findings suggest that this minimally invasive approach may have potential as an adjunct to conventional periodontal therapy; however, the present case cannot establish histologic periodontal regeneration or true new attachment. Controlled clinical studies with longer follow-up and appropriate regenerative endpoints are required to determine its efficacy and biological effects.
